# Energy-level alignment at interfaces between manganese phthalocyanine and C_60_

**DOI:** 10.3762/bjnano.8.94

**Published:** 2017-04-25

**Authors:** Daniel Waas, Florian Rückerl, Martin Knupfer, Bernd Büchner

**Affiliations:** 1 IFW Dresden, P.O. Box 270116, D-01171 Dresden, Germany

**Keywords:** C_60_, manganese phthalocyanine (MnPc), organic interfaces, photoelectron spectroscopy (PES)

## Abstract

We have used photoelectron spectroscopy to determine the energy-level alignment at organic heterojunctions made of manganese phthalocyanine (MnPc) and the fullerene C_60_. We show that this energy-level alignment depends upon the preparation sequence, which is explained by different molecular orientations. Moreover, our results demonstrate that MnPc/C_60_ interfaces are hardly suited for application in organic photovoltaic devices, since the energy difference of the two lowest unoccupied molecular orbitals (LUMOs) is rather small.

## Introduction

Within the last decades we have witnessed considerable progress in the development and understanding of organic (opto-)electronic devices [1–10]. A key issue in any device is the energetics at the device interfaces as it determines charge transport across or charge separation at the corresponding interface [11–12]. Thus, it is not surprising that the investigation of organic semiconductor interfaces has a rather long history, and a large number of studies has resulted in an advanced understanding of such interfaces [11,13–25]. A significant step forward was achieved recently by the development of a theoretical framework which is able to reproduce previous experimental results and to provide a comprehensive overview over the possible energy level alignments [26].

One class of materials that has been considered as constituents of organic devices quite often is the family of transition-metal phthalocyanines [27]. Indeed, several phthalocyanine-based (model) devices have been realized [28–33]. In particular, organic photovoltaic cells containing, e.g., copper phthalocyanine (CuPc) can be found rather frequently in the literature [28,30,34–36]. There, the charge separation at interfaces between the phthalocyanine and an appropriate electron acceptor is a crucial process. Often, fullerenes (C_60_) and their derivatives are used as acceptor materials.

Amongst the transition-metal phthalocyanines MnPc is exceptional in some respects. Due to the participation of manganese 3d orbitals to the molecular electronic states close to the Fermi energy, MnPc differs significantly from other transition-metal phthalocyanines, as it is characterized by the smallest ionization potential, the largest electron affinity, the smallest band gap and the largest exciton-binding energy [37–42]. Furthermore, it has an unusual spin-state of the Mn^2+^ ion of *S* = 3/2 and shows a weak ferromagnetic interaction in the bulk [43]. In this respect, thorough studies of MnPc in comparison to other transition-metal phthalocyanines (e.g., CuPc) widens our knowledge and understanding of these systems and beyond.

In this contribution we present an investigation of the energy level alignment at MnPc/C_60_ interfaces using photoelectron spectroscopy (PES). We show that this alignment depends on the preparation sequence and that the lowest unoccupied molecular orbitals (LUMOs) of the two molecules lie energetically very close at the interfaces, which is disadvantageous for applications in organic solar cells.

## Experimental

We have carried out valence-band and core-level photoelectron spectroscopy studies of MnPc/C_60_ interfaces at room temperature. The preparation and the analysis chamber had a base pressure of about 3·10^−10^ mbar. For the measurements an X-ray tube XR-50-M with a monochromator Focus-500, a gas-discharge lamp UVS-300 and a hemispherical electron-energy analyzer PHOIBOS-150 (SPECS) were used. The energy scales were calibrated with the Au 4f*_7/2_* core level emission feature of the substrate at 84.0 eV binding energy and the Fermi cutoff (0 eV binding energy) in the valence-band region. Furthermore, the valence-band spectra were corrected accounting for contributions of He I_β_ and HeI_γ_ satellites, assuming they had the same shape, and intensities of 1.8% (He I_β_) and 0.5% (He I_γ_) of the He I_α_ signal with an energy shift towards lower binding energies of 1.87 eV (He I_β_) and 2.52 eV (He I_γ_), respectively. To obtain the correct secondary-electron cutoff a sample bias of −5 eV was applied. The total energy resolution of the spectrometer was 0.35 eV for XPS and 0.15 eV for the UPS measurements.

For our investigations a pre-cleaned Au(100) crystal, controlled by core-level PES spectra, was used as a substrate, on which the MnPc/C_60_ heterojunctions were prepared. The fullerene and manganese phthalocyanine films were grown step by step at room temperature via evaporation of the two materials from home-built evaporators. The film thickness was monitored by a quartz crystal microbalance and additionally determined using the intensity change of the Au 4f*_7/2_* core level peak according to the method established by Seah and Dench [44]. We have grown the interfaces under investigation by both deposition sequences, MnPc on C_60_ and vice versa. After each MnPc or C_60_ deposition step C1s, N1s, Mn2p and Au4f core-level and valence-band photoelectron spectra were measured in order to follow changes of the electronic structure and to determine the energy level alignment at the interfaces.

## Results and Discussion

**Figure 1:**
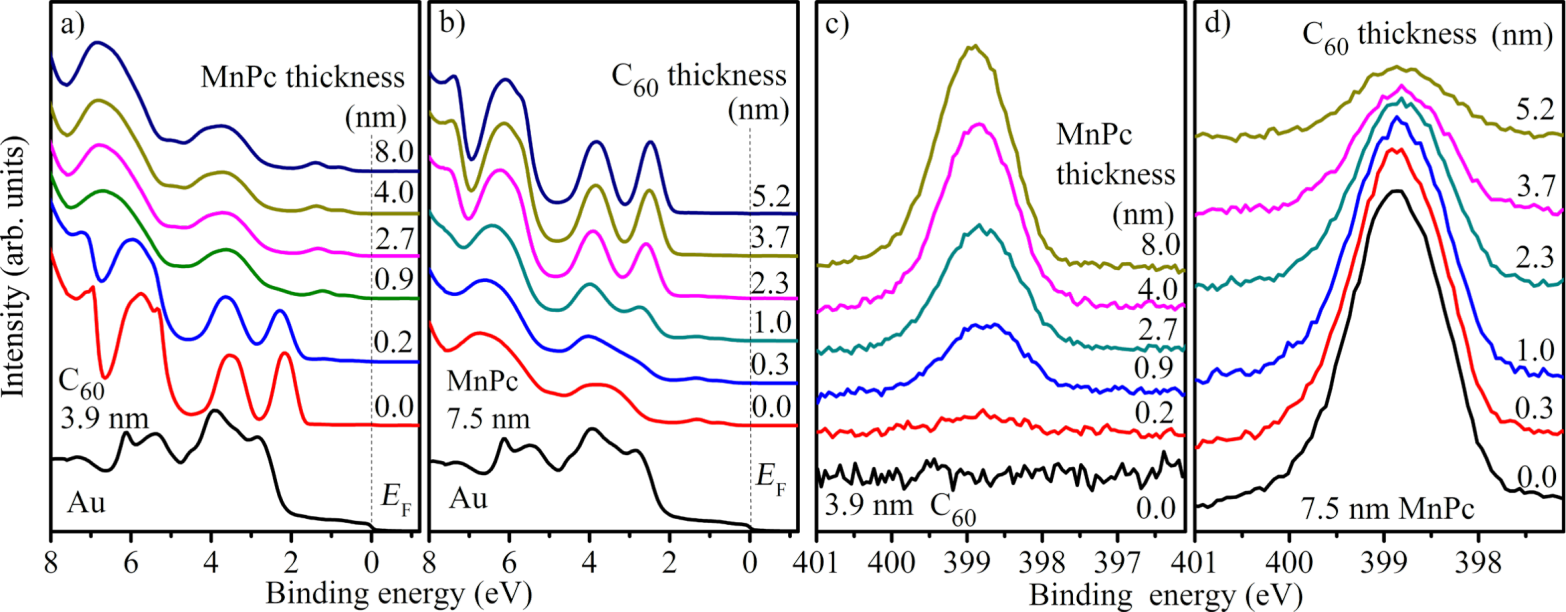
Evolution of the valence-band PES data (He I_α_) as a function of a) MnPc deposition onto C_60_ and b) C_60_ deposition onto MnPc. Additionally, the bottom spectrum represents the freshly prepared Au(100) surface. N1s core-level data of MnPc as a function of c) MnPc deposition onto C_60_ and d) C_60_ deposition onto MnPc. The corresponding layer thicknesses are indicated.

In Figure 1a and Figure 1b, we present the valence-band data as obtained from the freshly prepared gold substrate, from the starting layers of C_60_ and MnPc, respectively, and from the organic heterojunctions MnPc/C_60_ and C_60_/MnPc as a function of the respective layer thickness of the organic material on top. The corresponding layer thicknesses are displayed in these two figures. The spectra of pristine C_60_ and MnPc agree very well with those published earlier [39,41,45–49]. Upon deposition of the second organic material, the valence-band spectra change characteristically, the valence-band features of the second material, MnPc (Figure 1a) and C_60_ (Figure 1b), are observed and increase with increasing top-layer thicknesses until they are fully developed. In addition, there are energy shifts as a function of layer thickness, which indicate a change of the electrostatic potential at these interfaces as discussed below. There is no evidence for any additional contribution to the spectra and all spectra can be well described by a superposition of the spectra of pure C_60_ and MnPc. This clearly indicates the absence of chemical reactions at the interface studied here, as otherwise additional features or energy shifts would be expected.

We now turn to the discussion of the N1s core level data as obtained from the two deposition series, which are depicted in Figure 1c and Figure 1d. These data stem from MnPc only and, thus, allow insight into the behavior of one side of the interface under investigation. As a function of the corresponding overlayer thickness, the N1s core levels shift in energy, similar to what has been observed for the valence band data (see above). Apart from this energy shift, there are no significant changes in the measured line shapes except some broadening, which can be assigned to positional disorder right at the interface. This again indicates that the interface between C_60_ and MnPc is free of chemical reaction.

**Figure 2:**
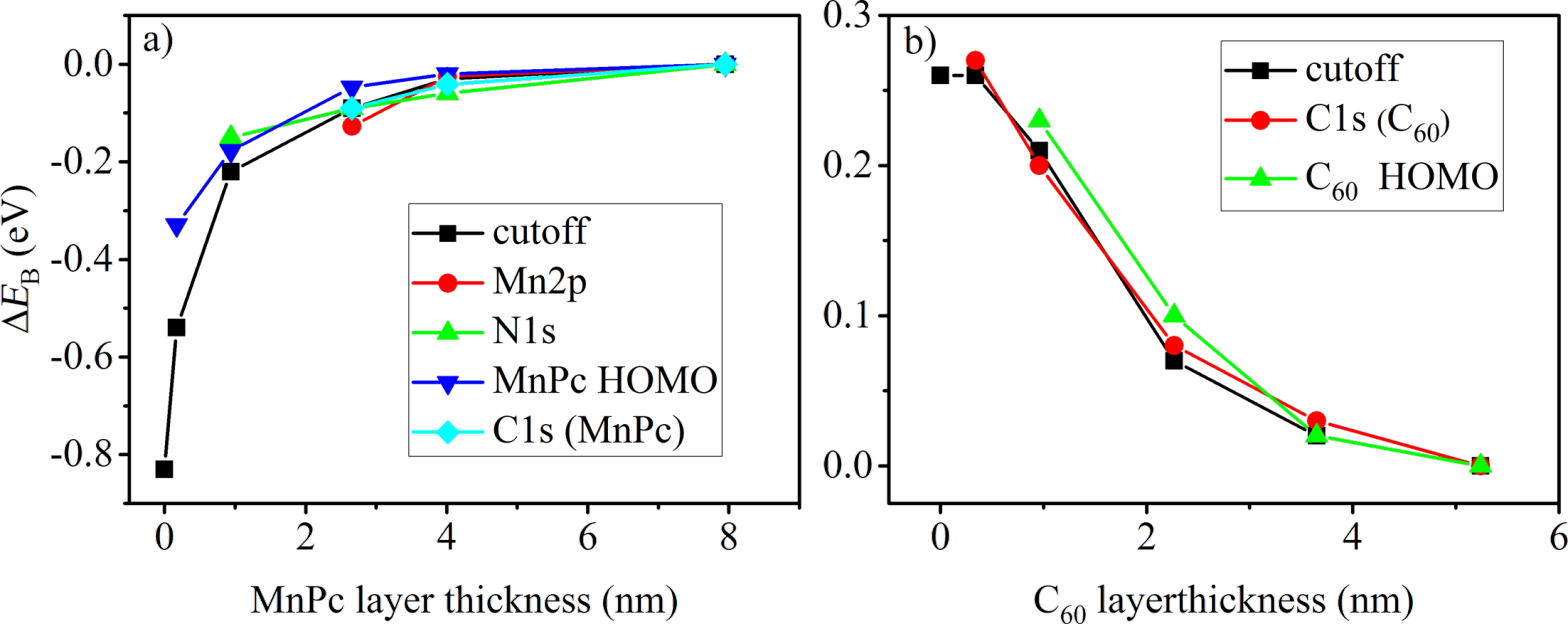
Comparison of the energy shifts of core levels, valence-band features and the secondary-electron cutoff (work function) of a) the C_60_/MnPc interface and b) the MnPc/C_60_ interface studied in this work.

Unfortunately, the information that can be obtained from the C1s core levels (see Supporting Information File 1) is less clear, since the contributions of the two materials overlap. We therefore have analyzed only the peak positions from those data sets in which the assignment to the corresponding MnPc or C_60_ structures is clear. Moreover, potential (energy) changes occurring at the interface can also be followed looking at the secondary-electron cutoff, which represents the work function of the actual sample (see Supporting Information File 1). In Figure 2 we summarize all the energy shifts that are observed in valence-band, core-level and secondary-cutoff data for both deposition series in a relative manner. Inspection of this figure makes clear that going across the MnPc/C_60_ interface, all available data shift in a very similar way. This is a strong indication for a common electrostatic potential for all electrons, in good agreement to our conclusions above that the MnPc/C_60_ is free from chemical interactions at the interface.

**Figure 3:**
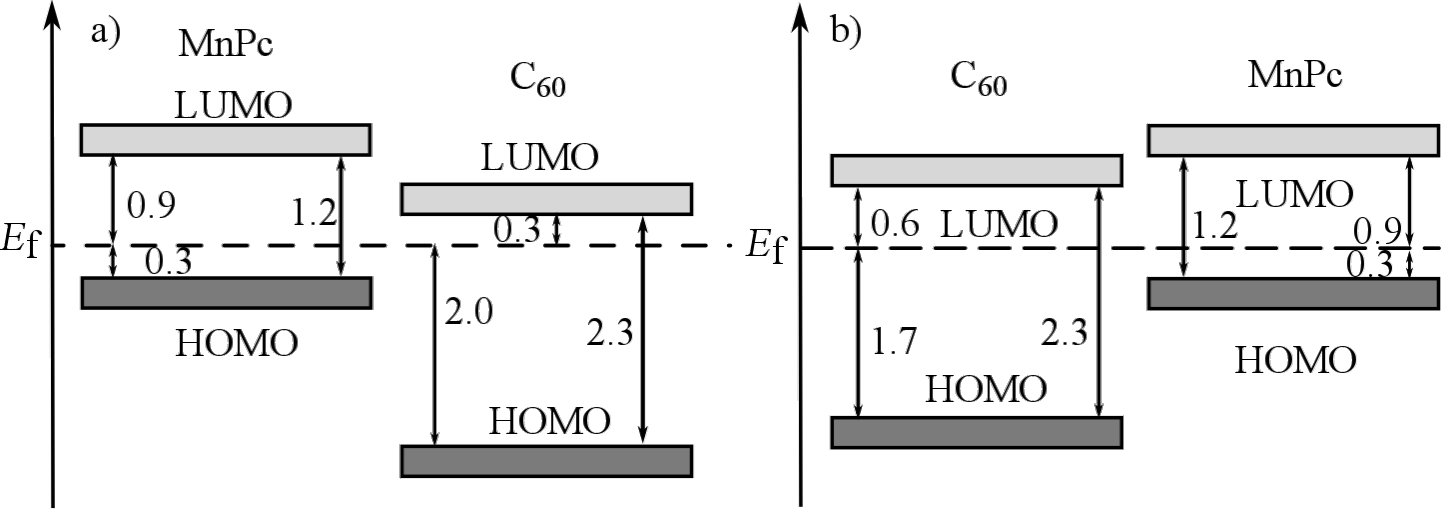
Schematic energy level diagrams of a) MnPc/C_60_, when C_60_ is deposited onto MnPc and b) C_60_/MnPc, when MnPc is deposited onto C_60_. All values are given in electronvolts.

From our data, we determined the energy-level alignments at the MnPc/C_60_ interface for both deposition sequences, which are shown in Figure 3. The according energies for the highest occupied molecular orbitals (HOMOs) have been taken from those thicknesses of the respective overlayers, for which the energy changes as seen in Figure 2 are virtually saturated. Moreover, we also included the energy position of the lowest unoccupied orbitals (LUMOs), which are derived taking into account the energy gap of the two materials (2.3 eV for C_60_ [46,50] and 1.2 eV for MnPc [41]). Figure 3 indicates a rather large offset of the HOMOs of more than 1 eV at the interface, while the energy positions of the LUMOs are much closer. These values are significantly different from those found for the interface between copper phthalocyanine (CuPc) and C_60_, where the HOMO offset was reported to be about 0.9 eV [51], while the LUMO offset can be estimated to about 0.8 eV. This difference is predominantly due to the rather different energy gaps in CuPc (about 2.2 eV [52]) and MnPc (1.2 eV). As a consequence, MnPc/C_60_ junctions are less suited for the application in organic photovoltaic devices since the energy gain associated with the charge separation at the interface is significantly reduced.

Interestingly, the energy-level alignment at the interface between MnPc and C_60_, prepared on a gold substrate depends on the deposition sequence. The HOMO offset differs by about 0.3 eV. Furthermore, the position of the Fermi level in MnPc is identical for the two cases (Figure 3). This is in contrast to the CuPc/C_60_ interface where the results were independent of the deposition sequence [51]. Following a recently introduced model [26], such a difference in the energy-level alignment would be expected, if the interaction of the organic layer deposited first and the metal substrate (gold) varies going from MnPc to C_60_ with the consequence of a different Fermi-level position in the layer stack. Moreover, in previous studies [25,53] the importance of interface morphologies, molecular orientations and the resulting density of states on the energy-level alignment has been demonstrated. For instance, at the interface between CuPc and F_16_CuPc a significant change in the ionization potential and work function due to the molecular orientation was observed [54]. Also, the orientation of phthalocyanine molecules has been used to influence the C_60_ energy levels at respective junctions [55]. It is further known that MnPc and other transition-metal-phthalocyanine molecules grow in a flat lying manner on Au(100) [49,56], while on top of C_60_ they exhibit an edge-on orientation (i.e., they stand up) [57–58]. This then can cause a different energy-level alignment as the ionization depends on the molecular orientation in the layers [53].

Finally, comparing our results to those from an associated measurement of co-evaporated mixtures of MnPc and C_60_ [59] one can find many similarities. The behavior of the valence-band features upon changing the mixing ratio was found to be equivalent to the observed changes with increasing the layer thickness as shown above. Relative energy shifts parallel the behavior as seen in Figure 3.

## Conclusion

We have determined the energy-level alignment at interfaces between MnPc and C_60_ using photoelectron spectroscopy studies. The relative energies at the interface depend on the deposition sequence. This is most likely a consequence of different growth modes of MnPc on either Au or C_60_ thin films. Moreover, our results show that at this interface the LUMO levels of MnPc and C_60_ lie energetically too close to render MnPc an appropriate absorber material in organic photovoltaic cells in contrast to, e.g., CuPc.

## Supporting Information

File 1 Additional spectra.
