# Supplementary material for: Energy-level alignment at interfaces between manganese phthalocyanine and C60
Source: Beilstein J Nanotechnol. 2017 Apr 25;8:927–32. doi: 10.3762/bjnano.8.94 (PMC5433147; doi:10.3762/bjnano.8.94)
Supplement: File 1 — Additional spectra. [file Beilstein_J_Nanotechnol-08-927-s001.pdf]

Supporting Information

for

Energy-level alignment at interfaces between manganese phthalocyanine and C_{60}

Daniel Waas*, Florian Rückerl , Martin Knupfer* and Bernd Büchner

Address: IFW Dresden, P.O. Box 270116, D-01171 Dresden, Germany

Email: Daniel Waas - d.waas@ifw-dresden.de; Martin Knupfer - m.knupfer@ifw-dresden.de

* Corresponding author

Additional spectra

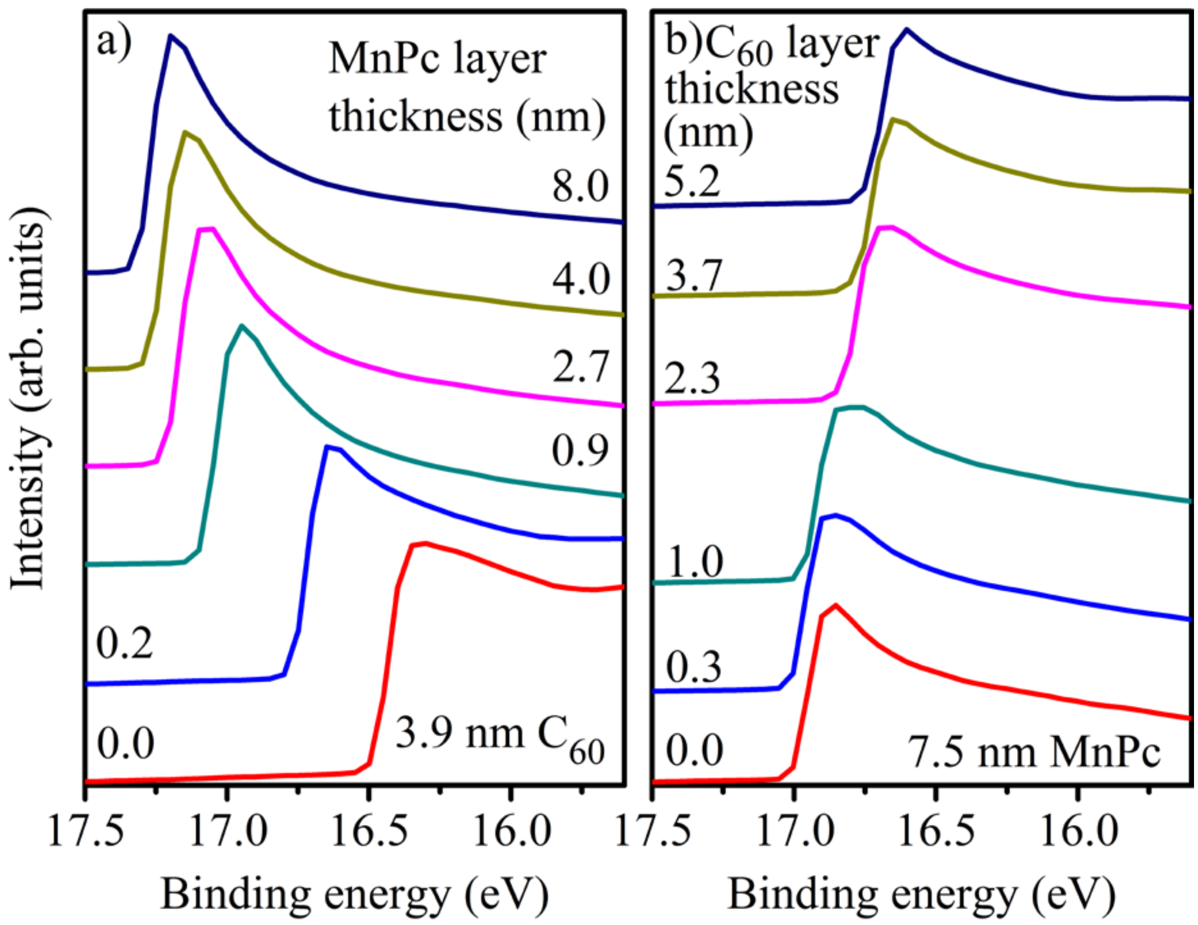

Figure S1: Evolution of the secondary-electron cutoff as a function of a) MnPc deposition onto C_{60} and b) C_{60} deposition onto MnPc, used to determine the workfunction. The corresponding layer thicknesses are indicated.

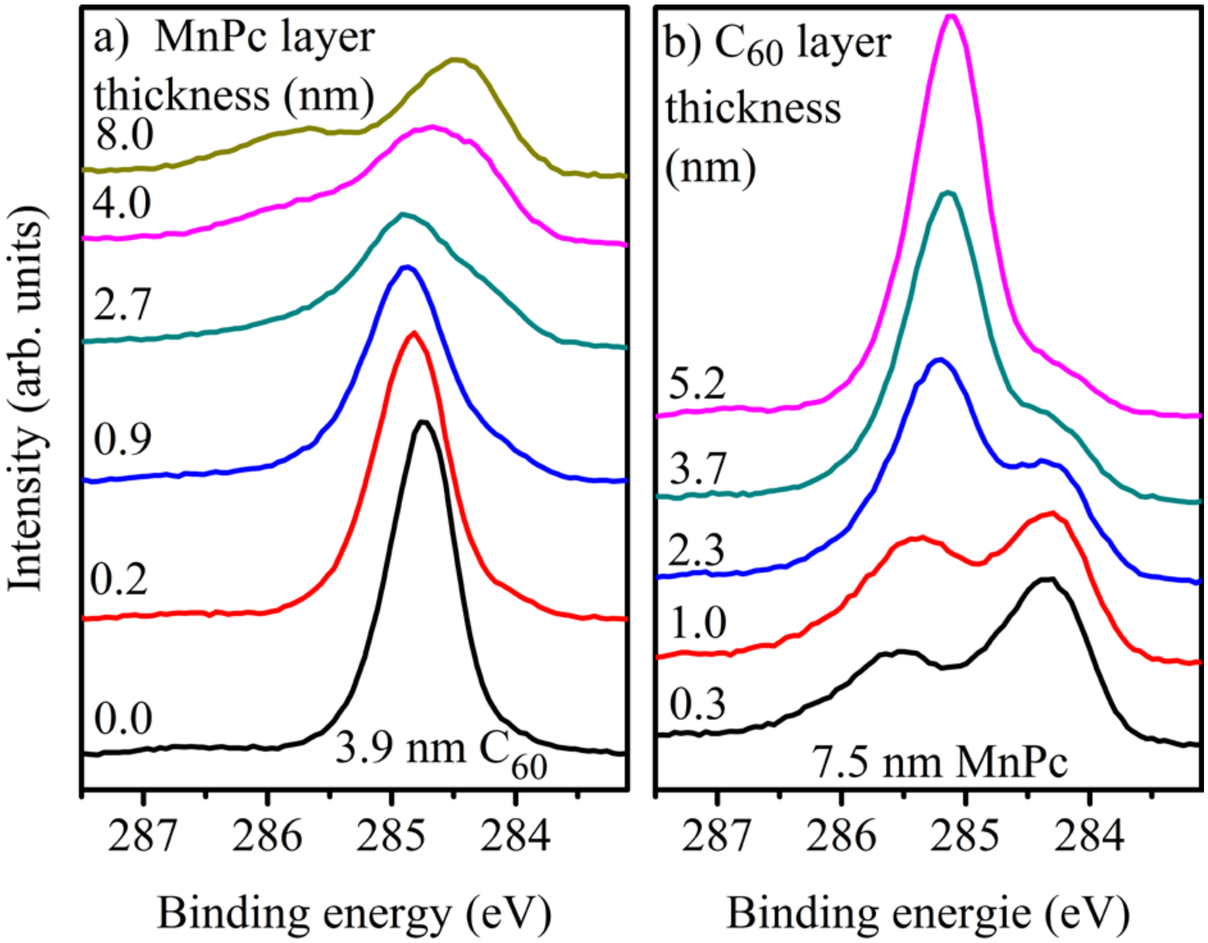

Figure S2: C1s core level data of MnPc and C 60 as a function of a) MnPc deposition onto C 60 and b) C_{60} deposition onto MnPc. The corresponding layer thicknesses are indicated.
